# Surgical Management and Therapeutic Strategy of Uterine Sarcoma According to Histological Subtype and Staging: Updated Review and Recommendations

**DOI:** 10.3390/cancers18121870

**Published:** 2026-06-08

**Authors:** Francisco Cristóbal Muñoz-Casares, José Gómez-Barbadillo, Rosa Álvarez-Álvarez, Nadia Hindi, Ana Sebio, Pablo Lozano-Lominchar, Juan Ángel Fernández-Hernández, Hugo Vasques, Paula Muñoz-Muñoz, José Manuel Asencio-Pascual

**Affiliations:** 1 Peritoneal Carcinomatosis and Retroperitoneal Sarcomas Unit, San Juan de Dios Hospital, 14012 Cordoba, Spain; joseangel.gomez@sjd.es; 2 Medical Oncology Department, Gregorio Marañón University Hospital, 28007 Madrid, Spain; ralvareza@salud.madrid.org; 3 Medical Oncology Department, Jiménez Díaz Foundation University Hospital, 28040 Madrid, Spain; nhindi@atbsarc.org; 4 Medical Oncology Department, Santa Creu i Sant Pau University Hospital, 08025 Barcelona, Spain; asebio@santpau.cat; 5 Surgical Oncology Unit, Gregorio Marañón University Hospital, 28007 Madrid, Spain; pablo.lozano@salud.madrid.org; 6 Surgery Department, Los Arcos del Mar Menor University Hospital, 30739 Murcia, Spain; juana.fernandez12@carm.es; 7 Surgery Department, Portuguese Institute of Oncology, 1099-023 Lisbon, Portugal; hugovasques@sapo.pt; 8 Surgery Department, La Fe University Hospital, 46026 Valencia, Spain; paumozmoz@gmail.com; 9 Hepatobiliary Surgery and Liver Transplant Unit, Gregorio Marañón University Hospital, 28007 Madrid, Spain; jmasencio@gmail.com

**Keywords:** uterine sarcoma, uterine mesenchymal tumors, leiomyosarcoma: endometrial stroma sarcoma, uterine adenosarcoma, undifferentiated uterine sarcoma, surgical management, surgical procedure, multimodal therapy, therapeutic strategy

## Abstract

Background: Uterine sarcomas represent a small and heterogeneous subgroup of uterine malignancies (accounting for less than 5%), characterized by high biological aggressiveness, high recurrence rates, and diagnostic complexity; surgical management remains the cornerstone of treatment and, consequently, the primary determinant of patient prognosis. Objective: This review synthesizes current evidence regarding the accurate diagnosis and appropriate surgical management of the main histological subtypes of uterine sarcoma across different stages, within a multidisciplinary therapeutic framework. Methods: A comprehensive narrative review was conducted using recent publications from major biomedical databases, with an emphasis on studies exploring surgical outcomes, molecular profiling, therapeutic strategies, and survival patterns. Results: Leiomyosarcoma and endometrial stromal sarcoma represent more than 80% of histological subtypes, with histological grade being the factor of greatest prognostic significance. Complete disease resection, without tumor fragmentation and with negative surgical margins, is the undisputed standard objective. Variations from the standard treatment—en bloc total hysterectomy with bilateral salpingo-oophorectomy—exist depending on histology and tumor staging. Conclusions: Individualized surgical treatment tailored to the specific histological subtype, combined with multimodal treatment strategies based on accurate tumor staging, molecular characterization, and recurrence patterns, is essential to improve survival. Such management should be conducted in referral centers with expertise in treating these rare and fearsome malignancies.

## Simple Summary

Uterine sarcomas are characterized by their rarity, biological aggressiveness, and high locoregional recurrence rates, showing poor response to conventional treatments and, consequently, dismal survival outcomes. Similar to other soft tissue sarcomas, initial surgery is a key factor in the prognosis of these patients. However, in most cases, patients receive incomplete or suboptimal surgery due to misdiagnosis at initial presentation. We present an updated review of the different histological subtypes of uterine sarcoma and the appropriate therapeutic strategy for each—specifically regarding individualized surgical procedures according to accurate tumor staging—with the aim of establishing clear and concise recommendations to improve the management and expectations of this malignant disease.

## 1. Introduction

Uterine sarcomas (US) account for less than 5% of all malignant uterine tumors. Originating from mesenchymal tissues such as the myometrium or endometrial stroma, they constitute a rare and heterogeneous group of neoplasms characterized by biological aggressiveness and poor prognosis. Advances over the last decades in the understanding of the disease have allowed for a better definition of their histological differences, immunohistochemical and molecular characteristics, and management specificities [1,2]. This review employs the terminology described by the WHO Classification of Tumours: Female Genital Tumours [3]. Consequently, we specifically address the five most frequent US subtypes, which represent 95% of the total: Leiomyosarcoma (LMS), low-grade endometrial stromal sarcoma (LG-ESS), high-grade endometrial stromal sarcoma (HG-ESS), undifferentiated uterine sarcoma (UUS), and uterine adenosarcoma.

The low frequency of these neoplasms, characterized by their intrapelvic location and non-specific clinical presentation that overlaps with very common benign pathologies such as leiomyomas (postmenopausal bleeding, pelvic pain, and increased abdominal girth), results in most cases being diagnosed following a total hysterectomy (TH) or myomectomy without prior suspicion of malignancy. This poses a significant risk for locoregional dissemination of the disease and the development of peritoneal implants. Furthermore, their tendency toward early and aggressive recurrence, combined with a limited response to conventional systemic treatments, renders them a profound therapeutic challenge finding within multidisciplinary tumor boards the basis for their appropriate clinical management [2].

The approach to gynecological sarcomas should follow the established recommendations for soft tissue and visceral sarcomas. In this regard, the objective of this review is to update current knowledge and recent literature to optimize their management. This begins with increasing diagnostic suspicion and improving histological identification and characterization at the time of diagnosis, ultimately enhancing surgical management—the fundamental pillar of treatment—always within the framework of a correct multidisciplinary therapeutic strategy aimed at offering patients the best care and prognosis for this disease.

## 2. Methodology

This work is based on an updated literature review aimed at synthesizing current and novel knowledge regarding the surgical management and multimodal treatment of uterine sarcomas. Given the rarity and heterogeneity of these tumors, as well as the predominance of retrospective series in the literature, a narrative synthesis was considered a valid and appropriate methodological approach.

A comprehensive literature search was conducted across PubMed/MEDLINE, Embase, Scopus, and the Cocrane Library, covering a study period from the publication of the new World Health Organization (WHO) terminology for uterine sarcomas in 2020 [3] through February 2026. Search terms included combinations of the following keywords and MeSH terms: “uterine sarcoma”, “uterine mesenchymal tumors”, “leiomyosarcoma”, “endometrial stromal sarcoma”, “uterine adenosarcoma”, “undifferentiated uterine sarcoma”, “surgical management”, “multimodal therapy”, “adjuvant therapy”, “hormonal therapy”, and “therapeutic strategy”. Boolean operators (“AND”, “OR”) were utilized to refine the search, and filters were applied to restrict results to human studies, the specified study period (2021–2026), and English-language publications.

Eligible publications included original research articles, retrospective and prospective cohort studies, clinical guidelines, consensus statements, systematic reviews, and meta-analyses. Case reports were excluded unless they provided relevant information on infrequent presentations or management considerations. Emphasis was placed on studies evaluating surgical outcomes, molecular profiles, recurrence patterns, and survival data across different histological subtypes.

The reference lists of key articles were manually reviewed to identify additional relevant studies. Data from the included publications were independently reviewed and qualitatively synthesized, with attention paid to consistent trends, areas of controversy, and gaps in the existing evidence.

This review was conducted without funding and no ethical approval was required, as it is a narrative review based exclusively on previously published data and does not involve human participants, patient data, or biological specimens.

## 3. Histological Classification and Epidemiology

US are rare uterine malignancies comprising a highly diverse histological group of tumors. These include leiomyosarcoma (LMS) as the most common subtype, followed by endometrial stromal sarcoma (ESS) with its two variants: low-grade (LG-ESS) and high-grade (HG-ESS). Other rarer subtypes include undifferentiated uterine sarcoma (UUS), adenosarcoma (AS), and extremely rare entities that will not be discussed in this review, such as rhabdomyosarcoma and tumors of indefinite malignant potential, such as perivascular epithelioid cell tumors (PEComa) and gynecological sarcomas with neurotrophic tropomyosin receptor kinase (NTRK) shift [3,4,5].

Carcinosarcoma derives from a monoclonal neoplastic cell and exhibits more characteristics of epithelial neoplasia than stromal neoplasia; consequently, it was excluded from the sarcoma group by the World Health Organization (WHO) in 2020 [3].

US generally demonstrate aggressive behavior, including the risk of distant metastasis even in early stages, and are mostly diagnosed between the fourth and sixth decades of life. They are associated with a poor prognosis in a significant percentage of patients, especially in the case of high-grade urothelial sarcomas [4].

### 3.1. Leiomyosarcoma (LMS)

Originating in the myometrium (uterine smooth muscle), it is characterized by high cellularity, marked nuclear atypia, elevated mitotic activity, and tumor necrosis. It is the most common subtype, representing more than 60% of US, with peak incidence in the sixth decade of life and a frequently unfavorable prognosis, as they are generally considered high-grade tumors with a high mitotic rate. The five-year overall survival rate is meager, with recurrence rates between 53% and 71% [6,7]. In a Surveillance, Epidemiology, and End Results (SEER) database report that included 13,089 cases in the US ranging 2000 and 2012, the five-year relative survival for the group of patients with uterine LMS was 42% [8]. Three main subtypes of LMS have been depicted: spindle, epithelioid, and myxoid [1].

Examples of the complex cytogenetic and molecular anomalies involved in its multifactorial etiopathogenesis include genetic alterations in TP53, RB1, and chromothripsis. The non-specific clinical presentation, similar to that of other benign diseases, along with the complexity of radiological and pathological patterns, hinders an early and accurate diagnosis [9]. In this regard, its primary differential diagnosis is with leiomyomas, extremely frequent benign tumors.

Between the benign entity—leiomyoma—and its malignant counterpart—leiomyosarcoma—there exists an uncommon uterine smooth muscle tumor with intermediate characteristics that warrants consideration: uterine STUMP (smooth muscle tumor of uncertain malignant potential), which requires strict follow-up due to the risk of recurrence, metastasis, or malignant transformation [10]. Occasionally, a preoperative diagnosis of uterine LMS may be confirmed as a STUMP after surgery. In other instances, a STUMP operated on years earlier may recur as LMS.

### 3.2. Endometrial Stromal Sarcoma (ESS)

ESS originates in the endometrial stroma, representing approximately 20% of US. ESS are predominantly intramural neoplasms that exhibit myometrial invasion and permeation of myometrial lymphovascular spaces. Based on their histopathological and molecular characteristics, alongside their differing clinical behavior, they are divided into low-grade (the most frequent) and high-grade [11].

#### 3.2.1. Low-Grade Endometrial Stromal Sarcoma (LG-ESS)

LG-ESS occurs in women between 40 and 55 years of age, of whom more than 50% are premenopausal. Although up to 25% are asymptomatic, the most frequent reason for consultation is vaginal bleeding, dysmenorrhea, or pelvic pain. Hormone receptors (estrogen, progesterone) are typically positive in the tumor cells. LG-ESS frequently present JAZF1 rearrangements. The prognosis for this histological variant of US is favorable compared to the rest, although recurrences can occur in up to one-third of patients, even after many years. The five-year survival rate for stage I–II is 90%, compared to 50% for advanced stage III–IV. Two-thirds of patients present with stage I–II at the time of diagnosis [7,12].

#### 3.2.2. High-Grade Endometrial Stromal Sarcoma (HG-ESS)

Abdominal bleeding, an enlarged uterus, or a pelvic mass at a mean age of 50 years (range 28–67) is the clinical presentation of HG-ESS. The tumor may appear as mural masses or intracavitary polyps. The mitotic rate is >10/10 HPF, and hormone receptors are negative [6,7]. Compared to patients with LG-ESS, those with HG-ESS experience more frequent recurrences that occur at shorter intervals following primary diagnosis. It is an aggressive tumor with a prognosis marked by an unfavorable median overall survival of 11 to 24 months, where up to 70% of patients are found to be in stage III–IV at the time of diagnosis [12].

### 3.3. Undifferentiated Uterine Sarcoma (UUS)

This is a malignant mesenchymal tumor that lacks specific, evident lines of differentiation in histological and molecular studies; therefore, it remains a diagnosis of exclusion. Generally, they exhibit a destructive pattern of myometrial invasion. They are very rare and aggressive tumors, representing approximately 5–10% of uterine sarcomas. Previously, they were classified into two subtypes: uniform or pleomorphic. These tumors may be associated with a low-grade stromal component and cyclin D1 expression. UUS typically consists of layers of uniform or pleomorphic epithelioid and/or spindle cells and also intense mitotic activity. They can show necrosis and lymphovascular invasion too [1]. Approximately 60% of patients at diagnosis present with stage III or IV disease. The prognosis is somber, with a median survival of less than one year once metastasis appears [7].

### 3.4. Adenosarcoma (AS)

It is characterized by a mixed structure of benign epithelial and malignant mesenchymal tissue that commonly resembles LG-ESS. Similar to UUS, it represents approximately 5–10% of uterine sarcomas. The mesenchymal component is high-grade, resembling HG-ESS, in 10–25% of cases [4]. AS with 25% or more pure sarcoma tissue is classified as AS with sarcomatous overgrowth. Most patients present with stage I disease (80–90%), with a 5-year overall survival of 60–80%. Survival is influenced by factors such as the presence of myometrial invasion, sarcomatous overgrowth, lymphovascular invasion, necrosis, and the presence of heterologous elements, including rhabdomyoblastic differentiation. The reported prevalence of sarcomatous overgrowth in patients with uterine AS is highly variable, ranging from 8% to 65%. Patients with sarcomatous overgrowth recur much more (23% vs. 77%) and present a reduced 5-year overall survival (50–60%) [7,13,14].

## 4. Diagnosis and Staging

### 4.1. Clinical Presentation and Laboratory Testing

Diagnosing uterine sarcomas can be challenging due to their rarity, numerous subtypes, and low diagnostic suspicion. In fact, there are no specific clinical or radiological criteria to differentiate uterine sarcomas from benign uterine leiomyomas. Signs and symptoms can be similar, such as abnormal vaginal bleeding, pelvic pain, and an abdominal mass. Uterine fibroids increase in volume by 20% to 30% annually during premenopause and spontaneously regress in approximately fifth of women. Subsequently, they stabilize or regress after menopause. Despite this, there is no consensus regarding the criteria for growth. Some authors have defined “rapid uterine or myomatous growth” as an increment comparable to 6 weeks of gestation in one year; others as a volume increase ≥20% every semester; and still others as a volume increase greater than 30% every three months during an observation period. However, a rushed growth of a uterine fibroid in a perimenopausal or postmenopausal woman should raise suspicion of sarcoma and a differential diagnosis process must be performed [1,15].

There are no specific serum tumor markers. Common gynecological markers (CA 125, HE4) are not useful as their results are unreliable. The systematic review by Cundari et al. [16] concluded that neither CA 125 nor HE4 can currently be considered definitive biomarkers for the diagnosis of uterine LMS. On the other hand, lactate dehydrogenase (LDH) is an enzyme present in almost all body tissues, and many conditions with high cell turnover (including cancer) can cause an increase in blood LDH levels. In this respect, some studies have shown a certain relationship between LDH elevations and some LDH isoenzymes with LMS, especially in combination with magnetic resonance imaging (MRI), but they have had no impact on clinical application [5]. Nor has the association observed in some studies regarding the combination of preoperative serum levels of LDH, D-dimer, and C-reactive protein, as an aid to distinguish LMS from uterine leiomyomas, especially in cases of degenerative or atypical myomas, had any impact to date [17]. In this sense, the recent systematic review and meta-analysis published by Taliento et al. [18], including 20 observational studies with a total of 13 biomarkers studied, shows the most encouraging results with LDH, but only to recommend further studies to confirm findings.

Interest has increased regarding the specific role of miRNAs in the diagnosis of uterine sarcoma, especially in recent years. miRNAs are small non-coding single-stranded RNA molecules that are able to behave as oncogenes or tumor suppressors under certain circumstances. Psilopatis et al. [19] highlight miRNAs as novel biomarkers that exhibit differential expression in uterine sarcoma cell lines and interact with certain genes related to tumorigenesis and cancer progression. Furthermore, some miRNA isoforms seem to be overexpressed or underexpressed in uterine sarcoma samples compared to normal or benign tumors. However, although the study results are promising for a possible standardized implementation, validation studies in larger cohorts are required.

### 4.2. Imaging Diagnosis

Initial radiological evaluation typically includes transvaginal ultrasound and combined pelvic-abdominal ultrasound, which may reveal a heterogeneous uterine mass, sometimes of large size, and at times accompanied by cystic areas, necrosis, and hypervascularization—all findings suggestive, though not definitive, of malignancy. Pelvic MRI provides greater tissue characterization and offers better data regarding local extension through algorithms with diffusion-weighted sequences that improve the differentiation between atypical leiomyomas and US [20]. The study by Raffone et al. [21], including prospective and randomized observational studies totaling 972 patients with leiomyomas or US, on the preoperative reliability of ultrasound, showed a sensitivity of 0.76 and a specificity of 0.89 for this diagnostic test. Another systematic review by Camponovo et al. [22], which included 12 ultrasound studies and 21 MRI studies, did not show that ultrasound had sufficient accuracy to diagnose sarcoma preoperatively, nor could it differentiate between the various subtypes. The key ultrasound features regarding uterine sarcoma were: solid tumor larger than 8 cm, indistinct borders, heterogeneous echogenicity, no acoustic shadowing, profuse irrigation, and internal cystic changes.

Results were mixed regarding MRI, with several studies showing high sensitivities in their analysis when combining multiple characteristics. In this respect, key features were: heterogeneity, irregular borders, high signal intensity on T2W1, hemorrhagic changes with an absence of central enhancement, hyperintensity on DWI, and low ADC (Apparent Diffusion Coefficient) values. Nevertheless, these findings need further verification in prospective studies with larger sample sizes. Recently, the meta-analysis by Woo et al. [23] aimed at analyzing the diagnostic performance of ADC values as part of multiparametric MRI, found a moderate to high combined diagnostic sensitivity and specificity for these ADC values in differentiating uterine sarcoma from leiomyomas.

Table 1 shows characteristic findings of different parameters evaluated by ultrasound and magnetic resonance imaging that help differentiate benign leiomyomas from leiomyosarcomas, although they lack definitive preoperative value. Ultrasound is the initial tool due to its accessibility and could serve as a screening diagnostic method. However, MRI is considered the technique of choice due to its better diagnostic accuracy and sensitivity and specificity exceeding 90% [22,24].

Computed tomography (CT) findings offer lower diagnostic specificity compared to MRI, often showing a large cystic-solid pelvic mass with irregular morphology and indistinct borders, occasionally involving adjacent structures [25]. However, contrast-enhanced CT is key for staging and allows for the evaluation of locoregional extension and distant metastasis.

Regarding fluorine-18-labeled fluorodeoxyglucose positron emission tomography (FDG-PET), it can be useful for indeterminate lesions or to distinguish malignancy in selected cases. Numerous studies have analyzed its utility in uterine sarcomas; however, most of them only reported on the utility of FDG-PET in diagnosing disease recurrence [22].

### 4.3. Histopathology and Molecular Study

Determining the benign versus malignant nature of a uterine lesion prior to surgical intervention is a priority for any uterine neoplasm requiring treatment, especially when conservative management is considered. Indeed, the absence of guaranteed benignity would necessitate definitive surgery rather than a conservative or fragmented approach.

Furthermore, when a uterine sarcoma is diagnosed, identifying the specific histological subtype is also a crucial preoperative objective, as it dictates the type of surgical procedure to be performed. Among the options for preoperative histopathological classification of uterine sarcomas are diagnostic curettage, hysteroscopic endometrial biopsy and ultrasound-guided needle biopsy. Although leiomyosarcoma (LMS) lesions are primarily localized within the myometrium, endometrial stromal sarcoma (ESS) and adenosarcoma (AS) may present as intrauterine polypoid masses. As a result, the positivity rate of diagnostic curettage for LMS is only 42.9%, whereas for ESS it can reach 83.3% [26].

Under these circumstances, the lack of diagnostic certainty with imaging and the potential for inadequate surgical management of an unnoticed malignant lesion—with its dire consequences—have led to the development of ultrasound-guided transuterine core needle biopsy or preoperative Tru-cut biopsy for myometrial lesions. In this respect, although the Tru-cut biopsy has only been validated by a few studies for its application in the diagnostic algorithm for uterine neoplasms, it is being widely used in gynecologic oncology for the treatment of pelvic and abdominal tumors. In addition, when combined with imaging techniques, its diagnostic accuracy and low complication rate guarantee the acquisition of sufficient material for the histological and molecular analysis of unclassified lesions. This facilitates treatment planning and reduces the diagnostic error rate in uterine sarcoma [27,28]. In this regard, intraoperative evaluation is discouraged; curettage and/or ultrasound-guided biopsies with coaxial needles represent the best and most reasonable alternative, even though their sensitivity for identifying US has not been considered definitive [4,29]. This is even more relevant following the recent publication by Smadja et al. [30], from the Bonvalot group at the Institut Curie in Paris. In a prospective cohort study, they analyzed the results of 34 patients with myomas and leiomyosarcomas diagnosed through preoperative percutaneous uterine core needle biopsy, microscopic examination, and comparative genomic hybridization (CGH) microarrays, which demonstrated a sensitivity and specificity of 100%.

From a pathological perspective, the challenging diagnosis of US will be based on an integrated histological, immunohistochemical and molecular evaluation. In fact, along with histological and immunohistochemical analyses, it is recommended to detect fusion transcripts and/or assess mutation status to refine tumor diagnosis and identify potential therapeutic targets using molecular tests [4,31,32]. Fluorescence in situ hybridization (FISH) and next-generation DNA and RNA sequencing are included in the assays that evaluate gene fusions. Based on its complexity, the diagnosis should be performed in referral centers with subspecialized gynecological pathologists experienced in uterine mesenchymal tumors and routine availability of molecular diagnostic techniques [4].

It is fundamental to conduct a meticulous study with extensive tumor sampling, following ICCR (International Collaboration on Cancer Reporting) guidelines, to allow for the evaluation of differential diagnoses such as sarcoma versus carcinosarcoma, LG-ESS versus HG-ESS, and STUMP versus LMS. Furthermore, for the definitive histological analysis, tumor fragmentation (such as intraoperative morcellation) must be avoided. Intraoperative rupture of the US, regardless of the risk of peritoneal dissemination, compromises sample integrity and the macroscopic evaluation of tumor size, as well as the interface between the tumor and the myometrium.

Table 2 shows the most characteristic findings of the main uterine sarcoma subtypes as assessed by ultrasound and pelvic MRI, along with the most significant histological and immunohistochemical findings [4,31,32].

### 4.4. Staging

Regarding the staging system for US, the FIGO (International Federation of Gynecology and Obstetrics) system, updated in 2009, is the most widely used. It encompasses the FIGO surgical staging system for leiomyosarcoma and endometrial stromal sarcoma, as well as for adenosarcoma. Table 3 illustrates this staging system, which enables treatment planning and prognosis prediction [1,33,34].

## 5. Treatment and Principles of Surgery

Surgical management is the cornerstone of treatment for US, and achieving complete resection of the disease without tumor fragmentation is the essential objective. Furthermore, the histological grade has a profound impact on the prognosis and clinical behavior. In this regard, the initial therapeutic approach decided by the multidisciplinary team will be based on prior information regarding the histological subtype and preoperative staging. Several studies have shown that patients treated at a sarcoma reference center, with better adherence to surgical recommendations, have higher recurrence-free survival [35,36].

Regarding minimally invasive surgical techniques, they increase the risk of uterine rupture during the procedure, with the corresponding intra-abdominal dissemination of the uterine sarcoma. Laparoscopic or robotic surgery is also associated with the frequent use of specimen morcellation, which can lead to a higher risk of recurrence and lower associated survival. Thus, expert groups and current clinical guidelines recommend that minimally invasive techniques should only be used by highly experienced surgeons who can ensure uterine integrity [1,4]. Regarding morcellation, even with strict protection measures using bags (contained morcellation), the consequences can be very negative if performed on a previously undiagnosed uterine sarcoma. Therefore, it is recommended to strictly avoid it in cases with a probable diagnosis of uterine leiomyoma but with risk factors for an occult uterine sarcoma: peri- or postmenopausal age, rapidly growing or new-onset leiomyoma, recent onset of symptoms such as abdominal pain or vaginal bleeding, previous pelvic radiotherapy, and/or exposure to tamoxifen [37,38].

### 5.1. Surgical Management in Early Stages

Surgical strategies for early-stage uterine sarcoma (US) are based on complete disease resection through en bloc total hysterectomy, with the uterine body and cervix removed entirely without tumor rupture and with negative surgical margins [39]. For US confined to the uterus (FIGO stage I), total hysterectomy is the standard treatment, while bilateral salpingo-oophorectomy (BSO) is generally performed in postmenopausal women. In premenopausal patients, BSO can be individualized, taking into account the higher risk of recurrence in those with LG-ESS or estrogen receptor-positive tumors [40]. The meta-analysis by Ronsini et al. [41], which included 14 retrospective comparative studies, did not show that bilateral oophorectomy significantly influenced overall survival (OS) or disease-free survival (DFS) in FIGO stage I uterine sarcomas. In FIGO stage II, the standard approach remains hysterectomy with BSO [4].

Systemic pelvic and para-aortic lymphadenectomy is not recommended, given the low incidence of nodal involvement in sarcomas confined to the uterus and the limited prognostic benefit of lymphadenectomy in US, except for the removal of macroscopically suspicious nodes. These were the conclusions of the meta-analysis by Si Manfey et al. [42], which align with a more recent meta-analysis by Li et al. [43] that included 32 retrospective cohort studies to analyze the impact of lymphadenectomy in uterine LMS and ESS. In the latter study, patients with uterine LMS or LG-ESS did not benefit from lymphadenectomy and might even experience worse overall survival (OS). However, patients with HG-ESS may indeed benefit from lymphadenectomy, in which case the best treatment is early, complete surgical resection with lymph node dissection. Nevertheless, large-scale population studies are required to obtain more robust data to validate these findings.

### 5.2. Surgical Management in Advanced Stages

In the largest multicenter study conducted in European countries to compare prognostic factors influencing the survival of patients with uterine sarcomas and their impact across all histological subtypes (SARCUT study), the achieved tumor cytoreduction was, along with histological type and FIGO stage, the primary prognostic factor in a cohort of 683 patients [44]. Given that the volume of residual disease is key to the prognosis of these US patients, the fundamental objective of the surgical approach is to achieve complete macroscopic cytoreduction (R0). In this regard, surgery for locally advanced stages may require total hysterectomy with BSO, lymphadenectomy of macroscopically suspicious nodes, and peritonectomy procedures with resection of infiltrated visceral organs, similar to the approach typically used in cytoreductive surgery for advanced ovarian cancer [4].

Advanced-stage uterine sarcomas are among the soft tissue sarcomas that most frequently cause peritoneal metastases. Most studies on uterine peritoneal sarcomatosis focus primarily on LMS and, less frequently, on ESS, as these represent the majority of cases. Consequently, the systematic review by Matsuzaki et al. [45] focuses on patients with peritoneal sarcomatosis from uterine LMS treated with CRS-HIPEC (radical cytoreductive surgery and hyperthermic intraperitoneal chemotherapy) and justifies the need for additional studies to examine the safety and effect of these procedures on survival in uterine LMS with peritoneal dissemination. In this sense, although the combined use of CRS-HIPEC is not part of the standard clinical practice for this type of peritoneal sarcomatosis, published studies on radical surgical treatment—typically performed by peritoneal oncologic surgeons—frequently include HIPEC as part of the procedure. Nevertheless, regardless of the unknown effect of HIPEC in these patients, radical surgical cytoreduction has achieved the best results in uterine sarcomatosis originating from LG-ESS and LMS, compared to the poor results obtained in HG-ESS and UUS, as shown in the series of 10 patients with uterine sarcomatosis undergoing CRS-HIPEC by Muñoz Casares et al. [46], reflecting the variable aggressiveness and prognosis of the different histological subtypes in advanced stages. These results are aligned with those from the series by Kusamura et al. [47] and Duzgun et al. [48].

**Figure 1:**
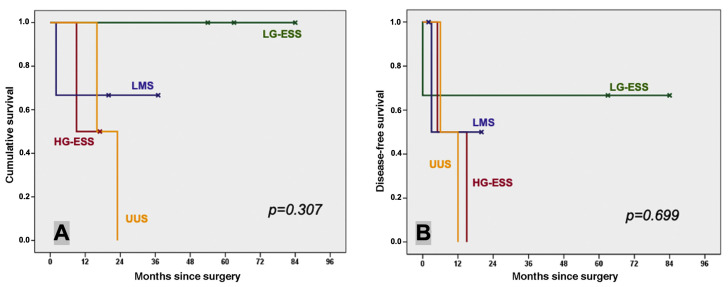
Kaplan–Meier survival curves in uterine sarcomatosis treated with CRS + HIPEC: (**A**) Overall survival (OS) according to histological subtype; (**B**) disease-free survival (DFS) according to histological subtype. Source: adapted from Muñoz Casares et al. Cir Esp. 2024; 102(8):433–442 [46]. LMS: leiomyosarcoma; LG-ESS: low-grade endometrial stromal sarcoma; HG-ESS: high-grade endometrial stromal sarcoma; UUS: undifferentiated uterine sarcoma; CRS: radical cytoreductive surgery; HIPEC: hyperthermic intraperitoneal chemotherapy.

Figure 1 displays overall survival and recurrence-free survival graphs for different histological subtypes of peritoneal sarcomatosis of uterine origin, surgically treated with CRS-HIPEC (Figure 1).

In the recent publication of the Ibero-American Consensus for the management of peritoneal sarcomatosis, participating experts reached a 98% consensus regarding the performance of CRS in patients with peritoneal sarcomatosis who had shown a response to induction treatment—particularly those with high-grade tumors—provided that options for complete cytoreduction existed. On the other hand, 88% of the experts acknowledged the limited evidence regarding the role of HIPEC after CRS in this context; consequently, they recommended its administration exclusively for patients who responded to induction chemotherapy and in whom complete cytoreduction was achieved, provided the procedure is performed in referral centers under clinical investigation [49].

The most representative series regarding the treatment of uterine peritoneal sarcomatosis through CRS and HIPEC procedures are shown in Table 4. These are retrospective series that clearly demonstrate that uterine LMS is the origin of sarcomatosis in the vast majority of patients [49,50].

Regarding FIGO stage IV, primarily in cases of distant metastasis, most studies are based on the management of metastatic soft tissue sarcoma and do not focus specifically on uterine sarcomas, although the findings are extrapolable.

If the tumor extends to other pelvic organs such as the bladder or rectum (FIGO stage IVA), the indicated surgery consists of a radical en bloc resection through pelvi-peritonectomy and extraperitoneal hysterectomy, with associated resection of infiltrated organs, following an adequate assessment by a multidisciplinary team. In patients with distant metastasis (FIGO stage IVB), the option of primary radical resection will depend on the number and location of the metastases, as well as the biology and histological subtype. Primary oligometastatic disease surgery should be considered, along with complete surgical resection of the primary tumor, if deemed feasible with acceptable morbidity. In cases of initially unresectable US, or resectable with unfavorable prognostic factors (multiple metastases with high tumor burden, systemic locations other than the lung or high risk for resection, high histological grade with extensive lymphovascular invasion, more aggressive histological subtypes with short free interval and rapid progression), primary systemic treatment is the recommended option, with subsequent re-evaluation for surgery based on the response [4].

In the Ibero-American Consensus for the management of liver metastases in soft tissue sarcomas (STB), oligometastasis was defined as metastatic STB with ≤5 metastatic lesions in ≤2 different sites, confirmed by morphological staging and additional imaging modalities and/or nuclear medicine, as appropriate, in which radical treatment of all lesions is possible either by surgical or ablative procedures [55].

The systematic review published by Tirotta et al. [56] analyzed the oncological and surgical outcomes of patients undergoing liver resection for sarcoma metastases in general, excluding GIST tumors, identifying the presence of synchronous extrahepatic metastases as a significant risk factor for lower overall survival. Other factors for a poorer prognosis were related to the size of the metastases, lack of response to chemotherapy, and a shorter disease-free interval. Furthermore, the study by Outani et al. [57], which analyzed the impact on survival in 84 patients with metastatic soft tissue sarcoma who underwent primary tumor resection and metastasectomy, showed a 5-year survival rate of over 25% (one-fourth of patients) and detected no significant differences between groups with synchronous and metachronous metastases.

Another systematic review focused on lung metastases from soft tissue sarcomas and published by Stamenovic et al. [58] included eight retrospective studies with more than a thousand patients with lung metastases who underwent metastasectomy. The most frequent histological subtype was LMS, and the 5-year survival rate ranged between 20% and 58%, observing better survival when there was only a single metastasis, a longer disease-free period, and an R0 resection was obtained.

The series recently published by Nobori et al. [59] and Adachi et al. [60] retrospectively analyzed the outcomes of lung metastasectomy in uterine malignancies of different histological origins, demonstrating the survival benefits that lung metastasis surgery can confer, as well as the negative prognostic influence of sarcoma compared to other uterine malignancies and the impact of different histological subtypes.

Surgical metastasectomies should always be previously discussed by a multidisciplinary tumor board at a reference center or sarcoma network, and should only be done in high-volume specialized centers with experience in these surgical procedures. It is important to heft the risks of surgery and the expected benefits. The main target of liver or lung metastasis resection in uterine sarcoma should be to achieve an R0 resection. The extent of surgery should be determined based on anatomical factors (location) and oncological principles (complete resection), prioritizing parenchymal sparing [55].

Table 5 summarizes the recommended surgical procedures for uterine sarcomas, based on histological subtype and early (I-II) or advanced (III-IV) FIGO stage at the time of diagnosis [1,4,5,31,40,49,55,61,62].

### 5.3. Special Considerations

#### 5.3.1. Fertility and Ovarian Preservation

Achieving a complete and adequate surgical resection is key to the final prognosis of patients with US. The primary objective is to ensure a definitive initial surgery through total resection. In this context, fertility-sparing treatment lacks high-quality data to guide best practices in US. Although it is theoretically possible and successful pregnancies have been described, it carries significant risks and long-term survival data are not available; therefore, it should only be considered in highly selected cases under strict follow-up protocols.

The meta-analysis published by Ronsini et al. [41], which included 14 retrospective comparative studies, did not show that oophorectomy significantly affected disease-free survival or overall survival in the treatment of FIGO stage I uterine sarcomas. Another systematic review published by Cucinella et al. [63] included nine retrospective cohort studies analyzing oncological and reproductive outcomes associated with fertility-sparing treatment in LG-ESS patients, showing a relatively high risk of tumor relapse, although it did not increase death risk, with an overall pregnancy rate of 41% and a live birth rate of 78%. In short, resection of the malignant uterine lesion combined with adjuvant hormonal treatment may be considered in selected early-stage patients with close follow-up.

In this regard, although applicable to all FIGO stage I histological subtypes, this conservative treatment is not recommended for HG-ESS or other aggressive subtypes due to their high recurrence rates and poor prognosis. Premenopausal women with uterine-confined LMS and negative hormone receptors constitute a subgroup of patients that warrants special consideration for fertility-sparing surgery, in those cases who desire it. Strategies for reducing estrogen levels such as bilateral oophorectomy or ovarian suppression with GnRH analogs should be considered in recurrent AS without sarcomatous overgrowth and LG-ESS in premenopausal women with ovaries in situ [1,4,5,32].

#### 5.3.2. Management After Incomplete Surgery Due to Initial Suspicion of Leiomyoma

In cases of unintended morcellation/fragmentation of an unsuspected US, the risks of recurrence are primarily concentrated in the pelvis and the development of peritoneal sarcomatosis. The use of a morcellator in cases of undiagnosed uterine LMS—as demonstrated by the meta-analysis by Bogani et al. [64] and subsequently confirmed by the MITO study [65]—is associated with a significant increase in the risk of overall recurrence, intra-abdominal recurrence, and death. In this regard, although some studies link morcellation to local recurrence but not necessarily to overall survival in morcellated pT1 uterine LMS [66], it is generally accepted that morcellation, tumor fragmentation, and myomectomy are associated with lower early survival in women with occult uterine LMS, with a recurrence risk three to four times higher at 3 years compared to en bloc surgery [40].

The subsequent management in these cases depends on the type of surgery performed:

- After complete surgery (hysterectomy): Surveillance or adjuvant chemotherapy is recommended [40].
- After incomplete surgery (myomectomy, subtotal hysterectomy): In the absence of signs of progression on postoperative imaging, completion of the surgery (total hysterectomy) is indicated, as recommended by the Clinical Guidelines developed by ESGO (European Society of Gynaecological Oncology), EURACAN (European Reference Network on Rare Adult Solid Cancer), and GCIG (Gynecologic Cancer InterGroup) [4]. Studies have identified sarcoma foci in the surgical specimen in up to 36% of these patients, even with negative prior imaging [67].
- In cases of incomplete surgery with early pelvic progression or peritoneal sarcomatosis: A multidisciplinary discussion is required to determine the neoadjuvant treatment, which will depend on the histological subtype and the presence of hormone receptors. Once a tumor response is confirmed, surgical treatment remains indicated if complete radical cytoreduction is feasible [40,49].

#### 5.3.3. Recurrent Disease

The quality of the initial surgery for sarcoma is a critical prognostic factor regarding both recurrence-free survival and overall survival. However, even with adequate surgeries, recurrence of high-grade sarcomas is very frequent. Nearly half of patients with recurrent US manifest with abdominal/pelvic recurrence, while the other half display with lung metastases, with a median interval of 18 months until recurrence after complete excision of the primary tumor [4].

Recurrent US must be evaluated by a multidisciplinary team to determine if surgery is a reasonable and feasible option for each specific subtype, with the primary objective being complete resection. Best candidates for surgery should be selected based on the following criteria:

- Tumor biology and histology.
- Location, number of lesions, and tumor burden.
- Recurrence-free interval.
- Performance status and patient perspectives.
- Severity of comorbidities and potential complications.
- Previous treatment received.

Preoperative chemotherapy may be offered in some histological variants. Regarding LG-ESS, cytoreduction can improve outcomes when combined with hormone treatment [12]. In this sense, recurrent AS without sarcomatous overgrowth, LG-ESS, and tumors with positive estrogen receptors could be treated only with endocrine therapy or combining an initial surgery followed by endocrine therapy, similar to the approach in recurrent LG-ESS. On the other hand, in indolent US such as LG-ESS, low-grade AS without sarcomatous overgrowth, and selected low-grade LMS, a reasonable option in cases of second or third recurrences would be surgical resection [4].

In cases of disseminated recurrence, the type of US is more likely to be high-grade. The possibility of an effective systemic treatment favors the strategy of administering 2–4 prior cycles to consider surgery for those selected cases that show an adequate response. Surgery should be considered if a complete resection can be achieved [68].

Complete surgical resection in cases of recurrent metastatic lesions of uterine LMS is associated with a better prognosis compared to chemotherapy and/or radiotherapy [40,69]. In this regard, complete surgery for recurrent US will always be the best option, following an adequate multimodal treatment strategy designed by the multidisciplinary team. Resection of lung or liver metastases can potentially be performed with low morbidity in the setting of recurrent disease [58].

## 6. Adjuvant and Multimodal Therapy

### 6.1. The Role of Systemic Chemotherapy

Systemic chemotherapy plays a very different role regarding low-grade sarcomas, characterized by a more indolent clinical course, versus high-grade sarcomas, which are characterized by high biological aggressiveness and a recognized tendency for systemic dissemination.

#### 6.1.1. Low-Grade Uterine Sarcomas

Regarding low-grade US, there is no solid evidence to support the routine use of chemotherapy in LG-ESS, even in advanced or recurrent stages. Simple observation is an option for FIGO stage I. In the absence of conclusive prospective studies, adjuvant hormone therapy can be recommended starting from FIGO stage II, as will be analyzed in another section.

Chemotherapy is reserved solely for exceptional situations of rapid progression, loss of differentiation, and refractoriness to hormonal treatment (including multiple lines), despite having limited results. In this regard, chemotherapy is not a therapeutic standard, and its use should be evaluated on an individual basis within multidisciplinary committees and, whenever possible, in the context of clinical trials [4].

#### 6.1.2. High-Grade Uterine Sarcomas

Adjuvant chemotherapy in patients with high-grade US is not the standard of care, and few data are available in this regard. However, although not recommended for FIGO stage I, it could be considered by the multidisciplinary team for FIGO stages II-III due to the poor prognosis.

Systemic therapy is the standard in the metastatic setting (FIGO stage IV). Thus, chemotherapy may be considered before or after local treatment with surgery/radiation, depending on prognostic factors such as extent, number and location of metastases, and the previous disease-free interval [1,4,5,31].

Table 6 presents a summary of the recommended chemotherapeutic agents based on current scientific evidence [1,4,49]. Table 7 summarizes the indications for chemotherapy treatment for the main histological subtypes of uterine sarcomas [4,5,31].

### 6.2. The Role of Adjuvant Radiotherapy

The role of radiotherapy in uterine sarcomas must be understood as histology- and grade-dependent, with a clearer benefit in terms of local control than in overall survival. While its use should be exceptional and highly individualized in low-grade uterine sarcomas, in high-grade sarcomas, it can form part of the multimodal treatment in selected patients with a high risk of locoregional recurrence. The only randomized study comparing external pelvic radiotherapy with observation in the treatment of surgically treated stage I and II uterine sarcomas was published in 2008 by Reed et al. [70]. The primary objective of the trial was to evaluate the reduction in the risk of local recurrence and determine if this translated into a survival benefit. The sample included patients with LMS, carcinosarcomas (currently excluded from the sarcoma group), and ESS. The number of patients with ESS was too small to observe any effect. In the subgroup of patients with LMS, the analysis showed no benefit. Better control of local recurrence was only observed in carcinosarcomas, although it did not correlate with improvements in overall survival.

#### 6.2.1. Low-Grade Uterine Sarcomas

Due to evidence based on retrospective designs with unclear data and long-term side effects of adjuvant radiotherapy, this treatment is generally not indicated. The ESGO/EURACAN/GCIG guidelines published in 2024 recommend that the indication for radiotherapy in LG-ESS be assessed on an individual basis in multidisciplinary committees only for patients in the recurrent or metastatic setting [4].

#### 6.2.2. High-Grade Uterine Sarcomas

Adjuvant radiotherapy showed no reduction in the recurrence rate of uterine LMS in the early stage in a recently published meta-analysis [71]. It could be considered as an alternative in locally advanced stages where surgery is incomplete or not feasible. On the other hand, some retrospective studies from the French Sarcoma Group did show a response in the form of local recurrence reduction and improvement in overall survival with the administration of adjuvant radiotherapy in patients with HG-ESS and UUS (stages I-III) [72]. In this sense, although radiotherapy is not usually a standard practice, it may be considered depending on factors that increase the risk of recurrence, such as tumor size, margin involvement, and/or the number of affected nodes removed.

Overall, radiotherapy can also play a palliative role in the control of symptoms of recurrent or metastatic disease when the quality of life is affected.

Table 7 presents a summary of the indications for radiotherapy in the different histological subtypes of uterine sarcoma based on tumor stage [4,5,31].

### 6.3. The Role of Hormone Therapy

The role of hormone therapy in uterine sarcomas depends fundamentally on the histological subtype and the expression of hormone receptors. Thus, hormonal therapies—including aromatase inhibitors, progestogens, and gonadotropin-releasing hormone (GnRH) analogs—represent an effective option for the treatment of certain histological subtypes of advanced uterine sarcoma, particularly low-grade endometrial stromal sarcoma (LG-ESS). It may also be considered selectively in hormone-dependent leiomyosarcomas and adenosarcomas, while it lacks indication in undifferentiated sarcomas and the majority of HG-ESS [73].

#### 6.3.1. Low-Grade Uterine Sarcomas

The rationale for this selective therapeutic option among different histological subtypes is based on the varying rates of estrogen receptor (ER) and progesterone receptor (PR) expression. LG-ESS is the subtype with the highest and most consistent hormone expression (70% ER and 90% PR) [73]. Positivity is usually strong and diffuse, explaining the high sensitivity to progestogens, aromatase inhibitors, and GnRH analogs. A comparative retrospective cohort study by Saab et al. [74] involving 221 patients with LG-ESS showed that ER and PR negativity is associated with poorer survival, reinforcing the clinical relevance of hormone expression.

Data regarding the use of GnRH agonists in LG-ESS are relatively scarce compared to the current knowledge on the efficacy of progestogens and aromatase inhibitors. Indeed, hormonal treatment has displaced chemotherapy as the first-line option for recurrence, locally advanced, or metastatic LG-ESS [75].

The second histological subtype with the highest hormone receptor expression is adenosarcoma (AS) without sarcomatous overgrowth (85% ER and 80% PR). In cases with sarcomatous overgrowth, hormone positivity decreases significantly (50% ER and 25% PR) and clinical behavior is more aggressive, limiting the benefit of hormone therapy [76]. Consequently, the indication for hormonal treatment in AS without sarcomatous overgrowth is similar to that of LG-ESS in patients with locally advanced, recurrent, or metastatic disease.

#### 6.3.2. High-Grade Uterine Sarcomas

Regarding uterine LMS, a tumor with marked intertumoral heterogeneity, hormone receptor expression is typically more frequent only in tumors with a low proliferative index and indolent clinical course; therefore, the receptor rate is clearly lower than in LG-ESS (25–60% ER and 35–60% PR) [73]. These hormone receptor-positive LMS cases could be sensitive to endocrine therapy, although this is not a standard treatment for this entity. In this respect, data on hormonal therapies in uterine LMS are more limited than for LG-ESS. In one of the few prospective phase II studies, Slomovitz et al. [77] reported progression-free survival rates at 12 and 24 months for nine early-stage patients; these were 100% for those receiving letrozole, compared to 80% at 12 months and 40% at 24 months for the observation group. Despite being promising, these results are too limited to be extrapolated to a broader population. Comparable considerations apply to advanced LMS expressing hormone receptors treated with letrozole, as shown in the study by George et al. [78].

In HG-ESS, characterized by aggressive clinical behavior and very limited hormone receptor expression, hormone therapy has no established role. Similarly, it is not indicated for UUS, where hormone receptor presence is virtually nil and aggressiveness is extreme [73].

Table 7 presents a summary of the indications for hormone therapy in the different histological subtypes of uterine sarcoma based on tumor stage [4,5,31].

### 6.4. The Role of Targeted Therapies and Immunotherapy

In recent years, advances in the molecular characterization and the tumor microenvironment have driven research into targeted therapies and immunotherapy as alternatives to conventional chemotherapy, although their clinical impact remains limited and dependent on the histological subtype.

#### 6.4.1. Targeted Therapies

The development of targeted therapies in uterine sarcomas has fundamentally focused on the inhibition of signaling pathways involved in angiogenesis, cell proliferation, and tumor survival. Among these, tyrosine kinase inhibition has demonstrated clinically relevant activity, especially in uterine LMS, the most frequent subtype.

Pazopanib, a multikinase inhibitor targeting VEGFR, PDGFR, and other receptors involved in angiogenesis, remains the primary targeted therapy used in clinical practice for pretreated uterine sarcomas [79]. The meta-analysis by Picozzi et al., published in 2026 [80], showed clinical activity of pazopanib in soft tissue sarcomas, depending on the histological subtype, primarily characterized by disease stabilization and limited progression-free survival, but with a manageable toxicity profile. The authors conclude that further research is essential to identify specific patient subgroups that may obtain greater benefit from pazopanib and to explore potential biomarkers that could predict treatment response.

Simultaneously, other lines of research have arisen, given the repeatedly reported alterations in the DDR (DNA Damage Response) pathway in LMS. In this regard, targeting this pathway is of great interest for the treatment of LMS. Trials are currently underway to test agents such as PARP inhibitors in LMS. A combination of treatments targeting multiple vulnerabilities will probably be necessary. When developing new targeted therapies for the treatment of LMS, it will be crucial to consider the potential toxicities of current drugs and the cost-effectiveness of implementing the new treatments [81].

Furthermore, new targeted strategies based on specific molecular alterations are being explored, such as the use of PARP inhibitors in combination with chemotherapy in LMS with DNA repair deficiencies, currently under evaluation in phase II clinical trials (combinations of olaparib–temozolomide and olaparib–trabectedin) [82].

#### 6.4.2. Immunotherapy

The incorporation of immunotherapy into the treatment of uterine sarcomas (US) initially generated significant expectations; however, clinical results to date have remained limited and heterogeneous. Immune checkpoint inhibitors, particularly those targeting PD-1/PD-L1—such as pembrolizumab and nivolumab—have demonstrated modest response rates in most studies, especially when utilized as monotherapy [83].

Various analyses of the immune microenvironment have shown that uterine leiomyosarcoma (LMS) typically exhibits a low tumor mutational burden and variable PD-L1 expression, factors that may explain the primary resistance observed with conventional immunotherapy. This resistance appears related to a lack of T-lymphocyte infiltration within the LMS microenvironment, which could be partially attributed to an enrichment of immunosuppressive M2 macrophages [81]. Nevertheless, specific subgroups of patients, such as those with a high mutational burden or favorable immunological characteristics, might benefit from this strategy, although current evidence remains limited. In this regard, ongoing studies involving HG-ESS and UUS continue to drive research in this field [84,85].

#### 6.4.3. Combined Therapeutic Strategies

Combined strategies represent an especially active line of investigation. The combination of immunotherapy with chemotherapy, targeted therapies, or dual immune blockade (PD-1 alongside CTLA-4) has demonstrated increased immune activation in preliminary studies; however, this has not yet translated into consistent clinical benefits for uterine sarcomas.

Isolated cases and very small series—including multimodal combinations—suggest that this approach could be effective in selected scenarios, but these findings require validation in larger prospective trials [86].

## 7. Multidisciplinary Approach

All International Clinical Guidelines recommend the care of patients with sarcomas in specialized reference centers with expert multidisciplinary teams to obtain the best results [87]. Among specific publications on uterine sarcomas, we highlight a retrospective study of 100 patients referred to the Institut Curie and recorded in the NETSARC database (the national reference network for sarcoma in France) [35]. This study confirms that despite the high frequency of suspicious clinical and radiological signs, a large proportion of women undergoing surgery for sarcoma receive treatment outside of expert networks. Consequently, preoperative biopsies were not performed in 65.6% of patients, and only 14.1% received their initial surgery at a specialized center. Achieving a complete and adequate surgical resection is key to the final prognosis of patients with US, and this study demonstrated that surgery performed outside the network was significantly associated with: Morcellation (32.9% vs. 0%; *p* = 0.036), which is strictly contraindicated in suspected or occult US; a lower rate of negative R0 margins (52.4% vs. 100%; *p* = 0.006) and poor compliance with surgical guidelines (28.3% vs. 72.7%; *p* = 0.013). Multivariate analysis identified treatment outside of reference centers without expert multidisciplinary teams as an independent negative prognostic factor for overall survival.

## 8. Discussion

Uterine sarcomas represent a heterogeneous and clinically aggressive group of rare gynecological neoplasms, in which surgical treatment remains the cornerstone in all histological subtypes and stages, representing the most important modifiable prognostic factor [4]. This narrative review integrates current evidence on diagnostic strategies, surgical approaches, and multimodal treatment sequences, highlighting fundamentally the decisive impact of the quality of the initial surgery and appropriate decision-making based on histology on oncological outcomes.

A consistent finding in large population studies, retrospective series, and analyses of expert networks is that complete en bloc resection without tumor fragmentation is the most relevant prognostic factor in uterine sarcomas, independently of the histological subtype. This observation coincides with data from reference networks for soft tissue sarcomas that show superior recurrence-free survival and overall survival when primary surgery is performed in experienced centers following specific principles for sarcomas [35,36,87]. The detrimental effect of tumor morcellation—whether intentional or accidental—has been repeatedly demonstrated and can be of dire prognostic consequences after intra-abdominal dissemination, especially frequent in uterine LMS due to its dreaded confusion with uterine leiomyoma [64,65,88].

One of the main clinical challenges that current evidence highlights remains the preoperative identification of uterine sarcomas, which continues to be limited by the absence of reliable clinical, serological, or imaging biomarkers. Although multiparametric pelvic magnetic resonance and diffusion-weighted magnetic resonance have improved diagnostic precision, no radiological characteristic by itself allows for reliably differentiating sarcomas from atypical leiomyomas [4,11]. In this context, image-guided core needle biopsy has emerged as a promising diagnostic tool in selected patients with indeterminate myometrial lesions, especially when performed in specialized centers with access to molecular diagnostics. Although it has not yet been universally adopted, recent prospective data suggest that this strategy can significantly reduce the rate of accidental morcellation of sarcomas and allow for adequate surgical planning [27,28]. Possible controversies in the type of surgical approach should converge on a common principle: if there is no diagnostic guarantee of benignity, morcellation should not be performed, nor any surgical procedure that carries a risk of tumor rupture. Nonetheless, the possible advantages of a minimally invasive procedure cannot be ignored and should be considered in those cases performed by surgeons with great experience who can ensure the integrity of the uterus [1,4,89,90].

From a surgical point of view, this review reinforces the concept that treatment must be individualized according to the histological subtype and the FIGO stage. Although total hysterectomy remains the standard procedure in early-stage disease, the role of bilateral salpingo-oophorectomy and lymphadenectomy varies considerably. In LG-ESS, ovarian preservation may be considered in carefully selected premenopausal patients, although the risk of hormonal recurrence must be clearly weighed. On the contrary, in high-grade sarcomas and UUS, a more radical approach, including the evaluation of the lymph nodes, may be justified due to their higher metastatic potential [1,4,5,31].

In advanced-stage disease, complete macroscopic cytoreduction (R0) has been consistently associated with better survival, reflecting the principles established in soft tissue and ovarian cancer surgery. The available data suggest that aggressive surgical strategies, including multivisceral resections and peritonectomy, may be justified in selected patients, particularly in LMS and LG-ESS. However, results remain largely dependent on the histological aggressiveness, the biology of the disease, and the response to systemic therapy [1,4,5,31,40,49,55,61,62].

The role of cytoreductive surgery combined with hyperthermic intraperitoneal chemotherapy (CRS-HIPEC) in peritoneal sarcomatosis related to uterine sarcoma remains controversial. While retrospective series from specialized centers report acceptable morbidity and encouraging survival in carefully selected patients, the absence of prospective comparative studies prevents the formulation of firm recommendations. The current expert consensus supports CRS, while HIPEC should be considered experimental and limited to clinical research settings [49]. Nonetheless, the histological variability of uterine sarcoma could represent in the future a favorable factor regarding the possible recommendation of HIPEC in some specific histological subtype, which is yet to be demonstrated.

Adjuvant and multimodal therapies play a role that is clearly histology-dependent. Although adjuvant chemotherapy and radiotherapy have not demonstrated consistent survival benefits in early-stage LMS, they remain relevant in advanced or high-risk disease. In contrast, hormonal therapy has a well-established role in LG-ESS and in selected AS with hormonal response, often replacing chemotherapy due to its favorable toxicity profile and durable disease control. Emerging targeted therapies and immunotherapeutic strategies, while conceptually attractive, have thus far shown limited clinical efficacy, underscoring the need for molecularly stratified clinical trials [4,5,31].

It is necessary to recognize several limitations of the available literature. Most data stem from retrospective studies, institutional series, and expert consensus documents, reflecting the rarity of these tumors and the practical difficulties in performing randomized trials. Furthermore, heterogeneity in histological classification, surgical expertise, and adjuvant treatment strategies make direct comparison between studies difficult. Nevertheless, the consistency of key findings across independent cohorts reinforces the validity of current recommendations.

In summary, this updated review underscores that the optimal management of uterine sarcomas requires centralized care, early diagnostic suspicion, and histology-tailored surgical strategies within a multidisciplinary framework. Future advances are likely to depend on improved preoperative diagnostic tools, deeper molecular characterization, and international collaborative efforts to generate high-quality prospective data. Until such evidence is available, adherence to sarcoma-specific surgical principles and treatment in reference centers remains the most effective strategy to improve outcomes in this rare but highly aggressive disease.

## 9. Conclusions

The diagnosis and pathological classification of uterine sarcomas is a challenge, and treatment planning is key and must be multidisciplinary. Outcomes depend primarily on the histological subtype, tumor grade, and the adequacy of the initial surgical treatment, the latter being the fundamental pillar of uterine sarcoma management. Such circumstances recommend their management in specialized sarcoma reference centers. One of the most important aspects in the preoperative evaluation of these patients is to distinguish uterine sarcomas from benign uterine leiomyomas. Morcellation must be avoided whenever there are no guarantees of a benign nature.

Complete en bloc resection without tumor fragmentation and negative surgical margins in the first surgery represents the most consistent prognostic factor in all stages of the disease. The minimally invasive approach should only be considered if the integrity of the uterus and its complete resection can be ensured. In advanced disease, maximal cytoreduction—within a strategic multimodal therapeutic framework established by the multidisciplinary team—can significantly improve survival outcomes if the resection is a complete R0. The role of adjuvant therapies remains limited and depends on the subtype and tumor stage, being especially relevant in advanced and metastatic stages.

Advances in the genetic and molecular characterization of uterine sarcomas will allow us to better predict clinical behavior and will help us specifically guide global therapeutic management and surgical management in particular.

## Figures and Tables

**Table 1:** Findings in different parameters of diagnostic imaging tests (ultrasound and magnetic resonance) of uterine leiomyoma versus uterine leiomyosarcoma.

Diagnosis	Parameters	LEIOMYOMA	LEIOMYOSARCOMA
Ultrasound	Number	Multiple	Solitary
	Size	Variable	≥8 cm/fast growth
	Shape	Round	Oval/lobulated
	Echogenicity	Calcifications	Heterogeneous
	Fan-shaped shadowing	Frequent	Rare
	Irregular cystic central necrosis	Rare	Frequent
	“Cooked appearance”	No	Frequent
	Vascularity	Circumferential flow	Irregular, moderate to rich vascularization
Magnetic Resonance Imaging	Border	Clear	Irregular and nodular
	Enhancement	Varied	Irregular outline
	T1 weighted imaging signal intensity	Low	High signal in regions affected by bleeding
	T2 weighted imaging signal intensity	Reduced	Heterogeneous and intermediate
	Endometrial thickening	No thickening	Irregular
	Restricted diffusion	No limited	Restricted

**Table 2:** Findings in diagnostic imaging and anatomopathological tests in histological subtypes of uterine sarcoma.

Subtype Histological	Ultrasound Findings	Findings Magnetic Resonance Imaging	Main Histological Features	Characteristics Immunohistochemistry
LMS	Large heterogeneous mass distorting the uterine architecture and containing areas of necrosis and hemorrhage. Increased vascularization on Doppler ultrasound.	Large mass with irregular margins, intermediate to high signal intensity on T2-weighted images, and hyperintense areas on T1-weighted images. No central enhancement.	Bundles of spindle-shaped cells showing smooth muscle differentiation with moderate to severe pleomorphism. Tumor cell necrosis, marked cytological atypia, and ≥4 mitoses/mm^2^ (≥10 mitoses/10 high-power fields).	Positive expression of smooth muscle markers: desmin, h-caldesmone, and smooth muscle actin (SMA) and connective tissue markers such as vimentin.
LG-ESS	Typically, ultrasound identifies endometrial sarcoma (ESS) as an irregular, hypoechoic mass originating from the endometrium, with myometrial invasion. This finding can resemble other conditions, such as adenomyosis or degenerating fibroids. In some cases, the endometrium may also appear heterogeneous or exhibit cystic degeneration.	Endometrial mass with smooth or irregular borders. Myometrial infiltration is a common feature, especially in HG-ESS, which is more prone to hemorrhage and extensive necrotic areas. Unlike endometrial cancer, ESS often presents as multiple nodules infiltrating the myometrium.	Endometrial stromal tumor cells in the proliferative phase infiltrating the myometrium with or without lymphovascular invasion.	Strong expression of CD10, IFITM1, ER and PR, and focal positivity for cyclin D1. Genetic fusions (JAZF1-SUZ12, JAZF1-PHF1, EPC1-PHF1, MEAF6-PHF1).
HG-ESS			High-grade malignant endometrial stromal tumor with uniform, round and/or spindle-shaped morphology.	Positive for cyclin D1 and BCOR. YWHAE-NUTM2A/B fusions, ZC3H7B-BCOR fusions, or internal tandem duplications of BCOR.
UUS	Unreliable. It can lead to a misdiagnosis of uterine adenomyosis or leiomyoma.	Large, polypoid masses with a heterogeneous appearance and marked vascular and lymphatic invasion. Appearance similar to a bag of worms. Feather-like enhancement.	It lacks specific differentiation. Marked cellular pleomorphism and abundant mitotic activity with atypical forms.	In cases with compatible histology, immunohistochemistry (IHC) and molecular studies (RNA sequencing) are indicated to rule out other tumor types. YWHAE, JAZF1, and NTRK rearrangements may indicate rare pleomorphic sarcomas that should be excluded.
AS	Large polypoid mass within the endometrial cavity.	Multiseptate cystic mass of endometrial base with solid areas of low signal intensity on T2-weighted imaging.	Diagnosis based on morphology: phyllodes (leaf-like) architecture, cleft or dilated glands, lined by benign or ciliated endometrial epithelium and surrounded by a proliferative, typically hypercellular stroma.	Positive for CD10, ER, and PR (although these are usually negative in sarcomatous overgrowth). p53 “wild type” and low MIB1 proliferation index.

Leiomyosarcoma (LMS); low-grade endometrial stromal sarcoma (LG-ESS); high-grade endometrial stromal sarcoma (HG-ESS); undifferentiated uterine sarcoma (UUS); adenosarcoma (AS); immunohistochemistry (IHC); estrogen receptor (ER); progesterone receptor (PR).

**Table 3:** FIGO staging 2009 for uterine sarcomas.

Stage	Leiomyosarcoma and Endometrial Stromal Sarcoma	Adenosarcoma
I	Tumor limited to uterus	
IA	Less than 5 cm	Tumor limited to endometrium/endocérvix with no myometrial invasion
IB	More than 5 cm	Less than or equal to half myometrial invasion
IC	-	More than half myometrial invasion
II	Tumor extends beyond the uterus, within the pelvis	
IIA	Adnexal involvement	
IIB	Involvement of other pelvic tissues	
III	Tumor invades abdominal tissues (not just protruding into the abdomen)	
IIIA	One site	
IIIB	More than one site	
IIIC	Metastasis to pelvic and/or para-aortic lymph nodes	
IV	Tumor invades bladder/rectum or shows distant metastasis	
IVA	Tumor invades bladder and/or rectum	
IVB	Distant metastasis	

**Table 4:** Most representative series published with patients of uterine peritoneal sarcomatosis treated using CRS-HIPEC.

Author and Year	Design Study	Patients (n)	Primary Tumor Histology	HIPEC	PCI	CCS (%)	Morbidity G 3–4 (%)	Mortality 30 d (%)	DFS-5y (%)	OS-5y (%)	OS Median (Months)
Rossi 2004 [51]	Prospective (multicenter)	12 of 60	8 LMS, 4 ESS	doxo + cisplatin	mean 7.7 (2–21)	Overall CC0: 68 CC0-1: 100	Overall 23	0	ND	Overall 38	ND Overall 34
Kusamura 2004 [47]	Retrospective (single-center)	10	8 LMS, 1 ESS, 1 AS	80% doxo + cisplatin, 20% doxo + MMC	ND	CC0: 90 CC2: 10	0	0	30	65	ND
Baratti 2010 [52]	Retrospective (single-center)	11 of 37	11 LMS	doxo + MMC or cisplatin	mean 14.7 (2–34)	Overall CC0: 76 CC0-1: 84	Overall 21.6	Overall 2.7	ND median LMS 15 months	LMS 40 (best results)	LMS 29.5
Sardi 2017 [53]	Retrospective (multicenter)	36	29 LMS, 3 ESS, 3 AS, 1 other	22 doxo + cisplatin, 10 melphalan and 4 others: Cisplatin, MMC	median 16 (2–39)	CC0-1: 94	21	2.8	LMS 39 (<20 at 2 years in others)	Overall 32 (LMS 41, Others <29)	LMS 37
Díaz-Montes 2018 [54]	Retrospective (single-center)	26 (7 CRS + HIPEC, 5 no CRS; 14 CRS)	22 LMS, 2 ESS, 2 AS	melphalan	ND	CRS: 79 CC0; Group CRS+ HIPEC: 100 CC0	1 patient (20% Group CRS + HIPEC)	0	ND median group HIPEC 11.3 m; CRS 5.3 m	ND	CRS + HIPEC: 43.8; CRS: 35.9
Düzgün 2022 [48]	Retrospective (single-center)	8 of 22	5 LMS, 3 ESS	doxo + cisplatin	mean 12.8 (3–15)	Overall CC0: 73 CC0-1: 86	Overall 31.8	0	Overall 36	Overall 57	Overall 45.3
Muñoz-Casares 2024 [46]	Retrospective (single-center)	10 of 23	5 ESS, 3 LMS, 2 UUS	70% doxo + cisplatin, cisplatin, paclitaxel	median 17 (3–36)	Overall CC0: 87, CC0-1: 96	Overall 13	0	Overall 34.5 (US 34) (LG-ESS 67)	Overall 64.6 (US 56) (LG-ESS 100)	ND
Ray 2025 [50]	Retrospective (single-center)	16	7 LMS, 6 ESS, 3 UUS	62% doxo + cisplatin, 19% cisplatin + melphalan or cisplatin, melphalan	median 12 (6–20)	CC0:94	12	0	LMS 35, Overall 12 months	Overall 30 (LMS 33)	Overall 32, (LMS 38, ESS 20, UUS 16)

Abbreviations: AS: adenosarcoma; CCS: completeness of cytoreduction score; CRS: cytoreductive surgery; DFS-5y: disease free survival at 5 years; Doxo: doxorubicin; ESS: endometrial stromal sarcoma; HIPEC: hyperthermic intraperitoneal chemotherapy; LG-ESS: low-grade endometrial stromal sarcoma; MMC: mitomycin C; ND: no determination; OS-5y: overall survival at 5 years; LMS: uterine leiomyosarcoma; US: uterine sarcoma; UUS: undifferentiated uterine sarcoma. Source: Modified from Muñoz Casares et al. *Cancers* 2024, 16, 2646 [49].

**Table 5:** Recommended surgical treatment according to histological subtype and FIGO stage of uterine sarcoma.

Subtype Histological	Surgical Treatment (Early Stage)	Surgical Treatment (Advanced Stage)
LMS	Total hysterectomy ± bilateral salpingo-oophorectomy (ovarian preservation in selected cases: stage I and premenopausal). No lymphadenectomy if there is no macroscopic involvement.	Complete cytoreductive surgery (R0). In distant or unresectable metastases, assessment of complete cytoreduction options by a multidisciplinary team after prior chemotherapy treatment.
LG-ESS	Total hysterectomy with bilateral salpingo-oophorectomy (ovarian preservation in highly selected cases: stage IA and premenopausal women, after explaining the risks of recurrence to the patient). No lymphadenectomy if there is no macroscopic involvement.	Complete cytoreductive surgery (R0). In distant or unresectable metastases, assessment of complete cytoreduction options by a multidisciplinary team after prior endocrine treatment.
HG-ESS	Total hysterectomy with bilateral salpingo-oophorectomy. Consider lymph node dissection.	Complete cytoreductive surgery (R0). In advanced local or distant metastasis, assessment of complete cytoreduction options by a multidisciplinary team after prior chemotherapy treatment.
UUS	Total hysterectomy with bilateral salpingo-oophorectomy. Consider lymph node dissection.	Complete cytoreductive surgery (R0). In advanced local or distant metastasis, assessment of complete cytoreduction options by a multidisciplinary team after prior chemotherapy treatment.
AS	Total hysterectomy with bilateral salpingo-oophorectomy (ovarian preservation in selected cases without sarcomatous overgrowth, premenopausal patients, and stage IA disease). No lymphadenectomy if there is no macroscopic involvement.	Complete cytoreductive surgery (R0). Low-grade AS should be managed similarly to LG-ESS. High-grade AS should be managed similarly to HG-ESS.

**Table 6:** Summary of Recommendations for Systemic Treatment based on published Clinical Guidelines and Consensus.

Summary of Recommended Systemic Treatment in Advanced Uterine Sarcomas (Source: Modified from Muñoz Casares et al. *Cancers* 2024, 16, 2646) [49]	
US high-grade	√ LMS: The combination of doxorubicin and trabectedin should be the preferred first-line chemotherapy, especially when achieving a response is relevant. However, doxorubicin–dacarbazine and doxorubicin monotherapy are valuable alternatives. In patients who cannot receive doxorubicin, gemcitabine plus docetaxel is an option.
	√ Non-LMS histologies: doxorubicin monotherapy should be considered standard. However, anthracycline-based combinations (with ifosfamide or dacarbazine) are valuable options when achieving a response is relevant.
	√ Trabectedin, gemcitabine combinations (preferably with dacarbazine), and pazopanib are second-line and later options, depending on the first-line chemotherapy regimen received.
	√ Endocrine therapy with aromatase inhibitors may be a reasonable option for some patients with advanced uterine LMS with positive hormone receptors.
	√ Patients with NTRK-positive tumors should be offered an NTRK inhibitor.
US low-grade	√ Aromatase inhibitors (preferred hormonal therapy due to a better toxicity profile) or progestins are recommended as first-line treatment. Tamoxifen is contraindicated.

**Table 7:** Summary of Recommendations for the Systemic and Radiotherapeutic Management of the Main Histological Subtypes of Uterine Sarcomas.

Subtype Histological	Systemic Treatment	Radiotherapy	Other Considerations
LMS	Chemotherapy in advanced stages: -Non-standard in FIGO I -Consider adjuvant in FIGO II-III -Consider preoperative chemotherapy in FIGO III to improve surgical resectability -Standard in FIGO IV In low-grade metastatic disease with progression and positive hormone receptors, consider endocrine therapy	Not recommended in early stages. May be considered as adjuvant therapy for high-grade localized cancer and in recurrent or metastatic cases Alternative option if surgery is not feasible	Ovarian preservation is possible in premenopausal women with estrogen receptor-negative tumors confined to the uterus
LG-ESS	Hormonal therapy should be evaluated in cases of advanced, recurrent, and metastatic local disease	Not recommended. May be considered as palliative care in recurrent or metastatic settings	Hormone-sensitive tumor
HG-ESS	Chemotherapy in advanced stages: -Not standard in FIGO I -Consider in early stages in cases of high risk of relapse and morcellation -Standard in FIGO IV	It could be considered due to the risk of recurrence and in the palliative context of relapse or metastasis	High metastatic potential; some studies report that lymphadenectomy may improve survival. Consider pre- or postoperative chemotherapy for operated oligometastases
UUS	Chemotherapy in advanced stages and in cases of morcellation. Consider options similar to HG-ESS	It could be considered due to the risk of recurrence and in the palliative context of relapse or metastasis	Very poor prognosis; multimodal treatment is often required
AS	Non-standard in stage I Chemotherapy should be offered in cases of high-grade or overgrowth sarcoma (stages II, III, and IV) Consider adjuvant hormonal therapy in low-grade, hormone receptor-positive sarcoma (stages II, III, and IV)	Not recommended. Management of low-grade AS is similar to LG-ESS, and management of AS with sarcomatous overgrowth is the same as HG-ESS	Minimal metastatic potential; requires close monitoring if fertility preservation is desired

Abbreviations: AS: adenosarcoma; HG-ESS: high-grade endometrial stromal sarcoma; LG-ESS: low-grade endometrial stromal sarcoma; LMS: uterine leiomyosarcoma; UUS: undifferentiated uterine sarcoma.

## Data Availability

All the information analyzed is available in the bibliographic references or within the article itself.
